# Vitamin D in pituitary driven osteopathies

**DOI:** 10.1007/s11102-024-01439-3

**Published:** 2024-08-24

**Authors:** Sabrina Chiloiro, Flavia Costanza, Elena Riccardi, Antonella Giampietro, Laura De Marinis, Antonio Bianchi, Alfredo Pontecorvi, Andrea Giustina

**Affiliations:** 1 Dipartimento di Medicina e Chirurgia traslazionale, Università Cattolica del Sacro Cuore, Largo A. Gemelli, number 8, Rome, Italy; 2 Pituitary Unit, Department of Endocrinology and Diabetes, Fondazione Policlinico Universitario A. Gemelli, IRCCS, Rome, Italy; 3 Institute of Endocrine and Metabolic Sciences, San Raffaele Vita-Salute University and IRCCS San Raffaele Hospital, Milan, Italy

**Keywords:** Osteoporosis, Osteopenia, Fractures, Pituitary diseases, Neuroendocrine diseases, Bone health, Skeletal fragility, Vitamin D

## Abstract

The evidence that pituitary hormones may bypass peripheral endocrine glands to exert remarkable effects on the skeleton is gaining ground. Both hormonal excess and deficit may determine impairment in bone structure, and they commonly result in bone loss in patients affected by pituitary and neuroendocrine disorders. Vertebral fractures are the most common skeletal alterations and may occur independently of bone mass. Use of vitamin D (VD) supplementation is still debated in this setting. This review will focus on the interactions between different metabolites of VD and pituitary hormones, and the effects of VD supplementation on bone metabolism in patients with pituitary diseases.

## Introduction

Pituitary hormones, such as adrenocorticotropic hormone (ACTH), growth hormone (GH), thyroid-stimulating hormone (TSH), gonadotropins, and prolactin (PRL), regulate bone health and act also by maintaining the delicate balance of bone metabolism through direct and indirect actions mediated by peripheral hormones such as insulin-like growth factor 1 (IGF-I), cortisol, free triiodothyronine (fT3) and free thyroxine (fT4), estradiol, and testosterone. Both excess and deficit of pituitary hormones determine the impairment of bone quality and architecture, commonly resulting in different degrees of impaired bone mineral density (BMD), such as osteopenia, osteoporosis and increased risk of traumatic or spontaneous fractures. Vertebral fractures (VFs) are the hallmark of secondary osteoporosis in many pituitary disorders [1], resulting in a not-reversible complication of pituitary disease with reduced quality of life (QoL) [2].

The diagnosis and the management of bone frailty in patients with pituitary and neuro-endocrine disorders are still challenging. In fact, besides the possible use of bone active drugs, already approved for other forms of osteoporosis, which include anabolic treatments (i.e. teriparatide) and catabolic treatments (i.e. biphosphonates, denosumab) [3] it has been suggested that, given the high prevalence of vitamin D (VD) deficiency, VD supplementation can be useful in this specific setting. However, even if protective effects on fracture risk of the supplementation with VD have been showed in some populations with hypovitaminosis D (i.e., elderly, post-menopausal, glucocorticoid-induced osteoporosis), the preventive role on fracture risk of the supplementation with VD has been investigated only in few studies for secondary osteoporosis caused by pituitary and neuroendocrine diseases [2].

This review will focus on the interactions between different metabolites of VD and pituitary hormones, and on effects of VD supplementation on bone metabolism in patients affected by pituitary diseases.

## Methods

The literature search was performed in April 2024. We explored MEDLINE (PubMed database), using the following keywords: (osteoporosis OR osteopenia OR fractures) AND (pituitary diseases) AND (bone OR skeletal fragility) AND (vitamin D). The papers met the following inclusion criteria: (1) written in English; (2) published until April 1, 2024; (3) original studies and case reports regarding prevention and treatment of osteoporosis in pituitary and neuroendocrine diseases. We selected and analyzed pertinent articles and we focused on information regarding the management of skeletal fragility occurring in patients affected by pituitary and neuroendocrine disorders.

### The skeletal health in pituitary diseases: osteopenia, osteoporosis, and fractures

Impairment of bone structure and strength is a consequence of hormonal hypersecretion by pituitary neuro-endocrine tumors and hypopituitarism, which may be consequent to different pituitary diseases, such as hypophysitis [4], pituitary metastasis [5], craniopharyngioma [6], Langerhans cell histiocytosis [7], pituitary germinoma [8], and teratoma [9]. In patients with hyper- and hypo-secretion of pituitary hormones, an alteration of the bone structure may occur which causes loss of bone mass and skeletal fragility, with osteopenia, osteoporosis, and vertebral fractures. Osteopenia and osteoporosis are reported in 16–82% of patients affected by Cushing’s disease (CD) [10–12], in 35–80% of male patients affected by prolactinoma [13–15], in 30–54% of patients affected by hypogonatotropic hypogonadism (HH) [16]. As bone microarchitecture is affected in patients with pituitary disease, densitometry alone is considered not enough to evaluate bone health [17], requiring also morphometric vertebral assessment upon diagnostic completion [18]. VFs are reported in 11–76% of CD patients [10, 11, 19], in 30–60% in acromegaly patients [20–24] and in around 30% of patients affected by GH deficiency (GHD) [25].

### Hypovitaminosis D as a risk factor for osteopenia osteoporosis vertebral and non-vertebral fractures in pituitary disease

VD is a key hormone for the regulation of calcium/phosphorus and bone metabolisms. The role of VD has been studied mainly in skeletal health, but its action seems to have other targets, designating VD as a pleiotropic hormone [26]. Recent studies on VD highlighted relevant extra-skeletal effects also on muscles, respiratory system, immune system, and metabolism [27, 28]. In particular, several types of metabolic disorders, such as obesity, diabetes mellitus, insulin resistance, and hypertension, have been associated with low serum vitamin D levels [28, 29]. Biochemically, VD is an organic molecule derived from cholesterol, biologically inactive until it undergoes hydroxylations at two different sites, the liver and the kidney. After exposure to ultraviolet B (UVB) radiation from sunlight, 7-dehydrocholesterol is converted into cholecalciferol in the skin. The activation of VD is subject to precise homeostatic regulation to maintain normal levels of the circulating calcium [30].

Hypovitaminosis D is now extremely widespread in the world, for the progressive aging of the population and the lifestyle changing with lower sun exposure. It is the leading cause for rickets in children and osteomalacia in adults. Symptoms and signs of VD deficiency can be various and concealed, depending on the degree of hypovitaminosis D and on the age of subjects, therefore not always immediately identifiable [31, 32].

**Fig. 1:**
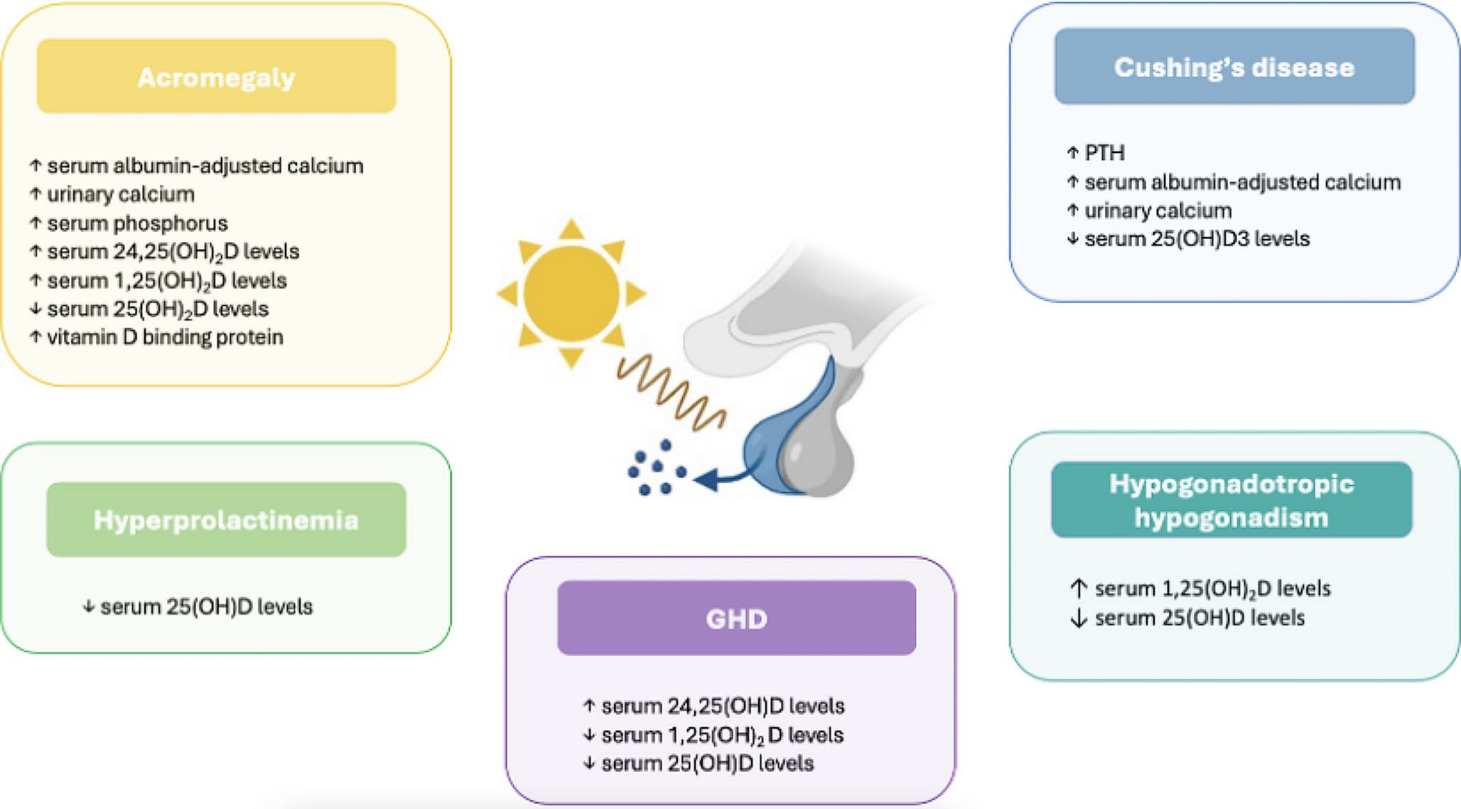
Schematic representation of the effects of pituitary hormone hypersecretion and deficit on calcium, D vitamin D metabolism.

**Fig. 2:**
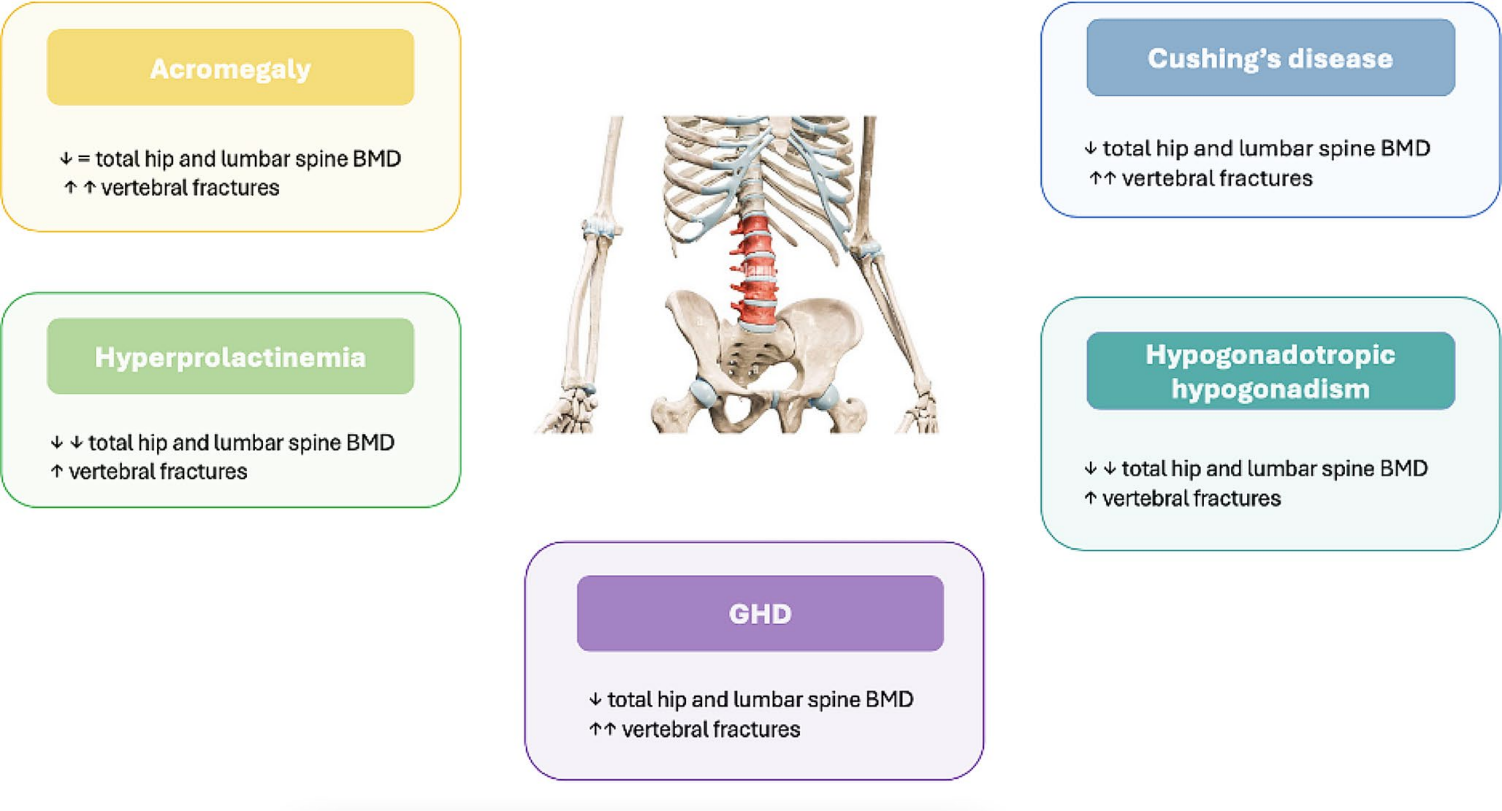
Schematic representation of the effects of pituitary hormone hypersecretion and deficit on bone metabolism and skeletal fragility.

The VD status impacts on the overall mineralization of the skeleton, bone turnover rate, and occurrence of frailty fractures. Epidemiological studies proved that the VD deficiency was associated with lower BMD, higher bone turnover and higher incidence of fractures: serum values of 25-hydroxyvitamin D (25(OH)D) less than 20 ng/mL were demonstrated to be associated with high risk of VD deficiency-related fractures [33, 34]. In parallel, an adequate VD supplementation increases the BMD, decreases the bone turnover, and reduces the incidence of frailty fractures [35–39], although results of the different trials are influenced from the different study-populations, doses, and type of tested VD formulations. A summary of VD metabolic and bone features in pituitary diseases is provided in Fig. 1 and in Fig. 2.

As in the general population the role of VD on the prevention of VFs in subjects with osteoporosis has been widely demonstrated [40], but few studies have investigated the effects of VD supplementation in patients with pituitary disorders.

### Vitamin D metabolism and levels in hyperfunctioning pituitary adenomas

#### Vitamin D in Acromegaly

Hypovitaminosis D is frequent in acromegaly patients [41]. The relationship between VD and GH/IGF-I excess is complex and until not fully clarified. Patients affected by active acromegaly usually present with hyperphosphatemia, hypercalcemia and hypercalciuria [42]. These alterations of calcium-phosphorus metabolism are mainly related to increased intestinal calcium absorption, induced by calcitriol (or 1,25(OH)D) and independent from the parathormone (PTH) action. Moreover, the elevation of 24,25(OH)D in acromegaly can be possibly due to the direct action of GH on renal 1 alpha-hydroxylase activity [43], influencing the renal metabolism of 25(OH)D, and modulating intestinal calcium absorption by stimulating the production of 1,25(OH)D [44]. Several studies reported high serum levels of 1,25(OH)D in acromegaly patients, particularly in individuals with biochemical active disease [42–51], suggesting that the excessive GH secretion may stimulate 1,25(OH)D production and directly stimulate calcium absorption [48]. However, the peripheral bioavailability of VD remains low in acromegaly patients. A direct effect of the liver microsomal enzyme system [45] and of the VD receptor (VDR) polymorphism have been hypothesized to regulate serum 25(OH)D concentration [45], according to the altered hormonal status [52]. Moreover, circulating levels of VD binding protein (VDBP) resulted increased in a study that compared 54 acromegaly patients with 32 control subjects [53], supposing that the IGF-I excess may modulate the VDBP concentrations via paracrine mechanisms [25].

In a study, that was conducted on a cohort of postmenopausal women with acromegaly, VFs were detected more frequently in patients with low total 25(OH)D levels [54].

Medical treatment in acromegaly seems to impact on VD levels: lipophilic substances malabsorption and fat-soluble vitamin deficiencies, such as VD, may occur during treatment with somatostatin receptor ligands (SRL): acromegaly patients treated with injective SRLs had lower 25(OH)D circulating levels, compared to not-treated patients [55]. Fiebrich and coauthors reported a 25(OH)D deficiency (defined as VD levels below 50 nmol/L) in 21% of 19 acromegaly patients treated with SRL for more than 18 months [56]. Otherwise, in another study, the serum 1,25(OH)D rose only during the short-term treatment (8 and 14 days) with octreotide, with no changes in VDBP [57].

In a study conducted on 16 acromegaly patients, the IGF-I normalization induced by the treatment with the GH receptor antagonist (pegvisomant) was associated with a reduction of serum 1,25(OH)D and increase of intact PTH levels, in absence of significant changes of 25(OH)D levels [58].

The use of dopamine agonists in the treatment of acromegaly is limited to patients considered affected by mild disease. Limited studies had evaluated the impact of these drugs on VD levels, but without conclusive results [43, 52, 59]. Finally, no studies have evaluated the effects of Pasireotide on VD homeostasis.

#### Vitamin D in Cushing’s disease

In CD, VD deficiency may be due to several mechanisms, such as the increased 25(OH)D catabolism, the reduced expression of specific calcium channels in the duodenum, the reduced intestinal calcium absorption, and the increased renal calcium excretion [60]. In fact, glucocorticoid excess is associated to hypercalciuria [61], and secondary hyperparathyroidism [62, 63], finally resulting in bone demineralization. For the loss of its antiadipogenic effect and the deregulation of the renin–angiotensin–aldosterone system, VD deficiency may contribute to the metabolic syndrome which usually manifests in CD [64]. A study assessed the effects of oral calcium load in patients with CD with and without osteopenia and showed higher serum VD levels only in subjects with osteopenia, probably due to an increase in PTH levels as for the effect of hypercortisolism [64]. The first studies that were performed on VD assessment in patients with hypercorticolism focused mainly on 25(OH)D and 1,25(OH)D, showing inconsistent and discordant results [65–67] on VD metabolites in CD and prevalently found no significant association between cortisol and VD levels. However, they were characterized by a great heterogeneity in the studied models (humans and animals), and only a few have been performed on endogenous hypercortisolism [66, 68–70].

Some experimental studies were performed on animals to investigate the impact of hypercortisolism on the expression of enzymes that are involved in VD metabolism, identifying an increased activity of the D-1alpha-hydroxylase and of the 24-hydroxylase, under glucocorticoid excess influence [71–74].

Povaliaeva and coauthors reported higher 25(OH)D/24,25(OH)D ratio in CD patients than healthy controls, suggesting a decreased activity of the 24-hydroxylase [75]. Guarnotta et al. proved an inverse correlation between serum 25(OH)D values and mean urinary free cortisol (mUFC) levels in a cohort of 50 CD patients [64]. Importantly, VD resistance has been proposed as a major feature in conditions of glucocorticoid excess [76]. Moreover, the effect of CD on VD metabolism may also be mediated by the suppression of GH caused by excess cortisol levels [77, 80].

There is still a total lack in the literature of studies regarding the treatment of Cushing’s syndrome and its correlation with VD levels, constituting a potential direction for future research.

#### Vitamin D and hyperprolactinemia

Both hyperprolactinemia and VD deficiency are associated with an increased risk of skeletal fragility [81]. PRL seems to exert direct effects on bone remodeling, depending on the circulating hormone levels, sex-hormones influence, and calcium supply. At physiological concentrations [82], PRL stimulates bone formation, whereas hyperprolactinemia stimulates bone resorption, with lower trabecular and cortical thickness, with this effect becoming more pronounced as PRL levels rise, with a progressive impairment of trabecular bone microstructure [2–83], and skeletal derangement [84, 85]. A recent study reported significant lower trabecular volumetric BMD, reduced trabecular number and trabecular thickness in patients with hyperprolactinemia, then in sex-matched healthy control, supporting the diagnostic role of the high-resolution peripheral quantitative computed tomography (HR-pQCT)in the investigation of skeletal fragility due to hyperprolactinemia [83].

Recent studies aimed to investigate the possible association between VD status and hyperprolactinemia, and some gender differences have been highlighted. A relationship between VD deficiency and elevated macroprolactin levels in premenopausal women has been identified [86]. Some studies evaluated 25(OH)D values in psychiatric patients with hyperprolactinemia, detecting deficient or inadequate levels of 25(OH)D in 37% of women [87]. Aboelnaga et al. found a higher prevalence of VD insufficiency/deficiency in a cohort of female patients affected by prolactinoma [88]. Krysiak et al. investigated the influence of VD status on prolactin response to treatment with cabergoline in a cohort of women with mild to moderate hyperprolactinemia (30–80 ng/mL), reporting that the decrease of PRL levels during treatment with cabergoline was more pronounced in patients with normal 25(OH)D levels than in women with VD deficiency or insufficiency [89]. In the same study, the authors reported that the HDL-cholesterol, triglycerides, hsCRP, fibrinogen, homocysteine, uric acid levels were proportional to the decrease in prolactin and baseline levels of 25(OH)D [89].

On the other hand, in a randomized, placebo-controlled pilot study, evaluating the supplementation with calcium and VD in young male subjects with risperidone-induced hyperprolactinemia, normal levels of VD were found [90]. In this case the hyperprolactinemia would be due to the antidopaminergic activity of the antipsychotics and could cause, in turn, hypogonadism and reduced bone mass. The mechanisms involved in the bone demineralization may differ between males and females. In women hyperprolactinemia impairs the pulsatility of gonadotropin-releasing hormone secretion, impairing the release of luteinizing hormone and follicle-stimulating hormone, and causing amenorrhea that can, in turn, lead to reduction in bone mass [90]. However, most studies did not define whether the evidence linking VD levels to prolactin was dependent or not of the presence of hypogonadism.

Despite the small patient cohorts analyzed, the female-only enrollment in most studies, the lack of prospective studies, which certainly requires greater in-depth analyses, these data seem to suggest a possible link between PRL and 25(OH)D, and that patients affected by hyperprolactinemia should often require VD supplementation.

### Vitamin D metabolism and levels in hypopituitarism

#### Vitamin D in growth hormone deficiency

GHD significantly influenced VD metabolism [91]. Low levels of 1,25(OH)D and 25(OH)D have been reported in children with GHD [92–94]. Moreover, insufficiency and deficiency of VD are reported in 27–50% of adult patients affected by GHD [95] with a gender difference in 1,25(OH)D levels suggested by Hitz et al., resulting significantly lower in female adult GHD patients [96].

GH replacement therapy with recombinant human GH (rhGH) may have a stimulatory effect on 1,25(OH)D levels. A significant increase in circulating VD levels was observed in 20 healthy male volunteers after 7 days of rhGH with a pharmacological dose [97], and in 7 healthy male volunteers after GH infusion [98]. Also, several studies showed an increase in circulating 1,25(OH)D concentrations in short (1 and 3 months) and long-term treatment (6 and 12 months) with rhGH replacement therapy, both in children [99–106] and in elderly adults [99] although a few other studies, in both children and adults, did not report significant effects of GH treatment on VD levels [107–110]. The data on the effects of rhGH replacement therapy on other VD metabolites such as 24,25(OH)D and 25(OH)D are not conclusive [103, 105, 106]. In studies conducted on hypophysectomized animal models, the renal conversion of 25(OH)D to 1,25(OH)D was markedly reduced, while the conversion of 25(OH)D to 24,25(OH)D was markedly increased. These data were confirmed by short rhGH treatment effects, resulting in a significant renal conversion increase to 1,25(OH)D and to 24,25(OH)D [107, 108]. These existing studies were strengthened by consistent data reported in studies on adult GHD patients. Independently from GH [109], IGF-I can also directly regulate the renal production of 1,25(OH)D by the 1α-hydoxylase [98, 110]. Conversely, also 1,25(OH)D can influence synthesis of IGF-I [111]. In animal models, a non-functioning VDR was associated with lower IGF-I levels compared with controls [112].

Soliman et al. evaluated VD metabolites in response to intramuscular administration of 300,000 IU cholecalciferol in 46 children with VD deficiency rickets and low growth velocity. After 6 months, the children showed increased height and growth velocity. In this study, a significant correlation was found between the increase in IGF-I and 25(OH)D levels, underlining that the activation of the GH/IGF-I system determined the accelerated linear growth after VD supplementation, and suggesting an important role of VD as a connection between the proliferating cartilage cells of the growth plate and IGF-I secretion, despite patients were not affected by GHD and therefore were not treated with rhGH [113]. A prospective study from Ameri et al. measured IGF-I levels after 12 weeks treatment with oral VD (5000 or 7000 IU/week) in 39 healthy adults. IGF-I levels significantly increased in the 7000 UI treated group, without any changes in the VD not-treated group [114]. Moreover, examining retrospectively 69 GHD patients on rhGH replacement therapy, higher IGF-I levels were identified in patients with higher 25(OH)D levels (≥ 15 ng/ml) [105]. These data were later confirmed by larger population studies [115, 116]. In parallel, normal VD levels are necessary to ensure optimal effects of rhGH treatment on bone quality in GHD patients. Several studies suggested rhGH treatment as a potential agent for attenuating or reversing the loss of bone mass in GHD patients [117, 118]. The correlation between the VD and IGF-I levels, and bone mineral density was demonstrated by several studies that proved that rhGH administration had a stimulatory effect on 1,25(OH)D [119] likely through enhanced activity of renal 1 alpha-hydroxylase through IGF-I action, without direct effects on the PTH, calcium, and phosphate [120]. In a prospective study including 57 adult GHD patients treated with rhGH for 2 years, an increase in the trabecular bone score was noted only in patients with 25(OH)D above the 50th percentile [117]. Although there is no consensus among the studies in the literature, it appears that rhGH therapy may influence VD metabolism, which could have a positive impact on skeletal health.

GHD patients may also have, as patients with acromegaly, significant cardiac comorbidities [121–123]. Impact of VD on cardiovascular risk in GHD was explored by Savanelli et al. This study involved 41 hypopituitary patients with GHD and 41 controls. Hypovitaminosis D seemed the most powerful predictor of the prevalence of dyslipidemia and hypertension [124]. Also the role of VD in the relationship between carotid artery intima-media thickness and IGF-I levels has been hypothesized in another study [125].

#### Vitamin D in hypogonadotropic hypogonadism

Male HH is a condition characterized by a decrease in serum testosterone levels due to a reduction in gonadotropin levels originating from the pituitary gland. Central hypogonadism constitutes a possible cause of secondary osteoporosis, which often requires, over sex hormone replacement therapy, the use of bone active drugs, adequate calcium intake and VD supplementation [126]. The simultaneous presence of secondary hypogonadism and hypovitaminosis D may contribute to increase the risk of skeletal fragility.

Interconnections between hormones of the hypothalamic–pituitary–testicular (HPT) axis and VD levels were demonstrated [127, 128]. Since the discovery of the expression of VD receptor and VD enzymes in human testis, ejaculatory tract and mature spermatozoa [129], a potential VD role in spermatogenesis has also been suggested. The presence of the enzymes CYP2R1, CYP27A1 and CYP27B1 in Leydig cells suggested that VD may also be connected to male reproductive hormone secretion. The VDR and 1a-hydroxylase (CYP27B1) are also expressed anterior pituitary and hypothalamus, as well as in the testicle [130, 131]. Data from the European Male Ageing Study (EMAS) suggested a link between 25(OH)D deficit and biochemical HH [132]. Also, Wehr et al. observed a positive correlation between testosterone and 25(OH)D levels, suggesting that serum VD may impact directly on gonadal function [133]. Another retrospective study reported a high frequency of VD deficiency (25(OH)D < 20 ng/mL) in patients with hypogonadotropic and hypergonadotropic hypogonadism, respectively 79% and 85% [16].

Very few studies have been performed on VD assessment in HH. A study investigated the metabolic effects of androgen therapy, detecting an increased availability on circulating levels of 1,25(OH)D in parallel with the increase in testosterone levels [134]. However, this study had the major limitation of the small cohort of 13 subjects with very limited statistical power.

### Vitamin D in other sellar lesions

Vitamin D metabolism and levels in patients with non-adenomatous pituitary diseases, such as hypophysitis, pituitary metastasis, craniopharyngioma and pituitary germinoma were not yet investigated. A single cross-sectional study from a national cohort of Dutch adult childhood cancer survivors, compared with population-level data from the Danish national registry, showed an increased risk of any first fracture and a very low lumbar spine BMD, associated with severe VD deficiency [135]. The authors recommended intensive surveillance and interventions for endocrine disorders and vitamin deficiencies to prevent further fractures in these patients.

### Effects of vitamin D supplementation on skeletal fragility in patients with pituitary diseases

Current guidelines recommend VD supplementation in patients with deficiency, although effects on the prevention of fragility fractures are still debated [135]. Responses to VD treatment depend strongly on the baseline level of 25(OH)D [136]. The VD supplementation results in substantial increases of 25(OH)D levels, normalization of calcium, phosphate and PTH serum concentrations, reduction in bone turnover, and increase in spine and hip BMD in patients with pre-treatment 25(OH)D lower than 30 nmol/L [35, 137, 138]. Biochemical improvement of VD metabolites was more moderate in patients with pre-treatment 25(OH)D levels > 30 nmol/L [35].

In patients at high risk for hypovitaminosis D (such as elderly, post-menopausal females) and in patients affected by glucocorticoid-induced osteoporosis, cholecalciferol supplementation is effective in the prevention of frailty fractures, despite data reported in general population [39, 139]. Some studies biases were identified, such as levels of VD at baseline, usage of different doses of cholecalciferol, or combination treatment with calcium and VD supplementation, and duration of follow-up [30, 140]. Instead, in a meta-analysis conducted on 12 double-blinded trials, VD supplementation resulted protective from the occurrence of nonvertebral fractures and hip fractures [141].

Although skeletal health is one of the most relevant clinical complications in acromegaly, a single study investigated the effect of VD supplementation in the prevention of VFs. In a recent multicentric and longitudinal study, conducted on 61 acromegaly patients, VD supplementation resulted protective on the occurrence of incidental VFs in a cohort of 61 acromegaly patients [142]. In this retrospective study, VD supplementation was administered in 26 patients, with pre-treatment V25OH-D lower than 20 ng/mL, according to the guidelines for treatment of VD deficiency [33] and the Italian reimbursement criteria in force during the study period. VD supplementation was found to be safe and effective in raising serum 25(OH)D levels, resulting also associated to lower frequency of development of incidental VFs. Parallelly, at the end of the study, the serum V25OH-D levels were lower in patients who developed i-VFs then in patients who did not develop i-VFs.

The correlation between VD levels and disease activity in acromegaly was confirmed in this study [142], as serum V25OH-D levels were higher in patients with biochemical controlled acromegaly then in those with active disease.

These data confirmed previous findings for which patients with acromegaly, particularly those with biochemically active disease, are at high risk of developing both VFs and hypovitaminosis D. A dysregulated VD metabolism was described in patients with active acromegaly, characterized by higher 1,25(ОН)D, lower 24,25(ОН)D and altered VDBP production, suggesting that the response to VD supplementation might be influenced by GH excess [143].

In two recent studies conducted on patients with active Cushing disease, the supplementation of VD with a load of 150,000 UI of cholecalciferol resulted in a significant reduction of serum PTH levels and in a significant increase of serum calcium, V25OH-D, V1,25(OH)2D and 24,25(ОН)D levels [64, 75].

## Conclusions

VD is a key hormone in the regulation of calcium/phosphorus metabolism and skeletal health. The deficit of VD in highly prevalent in the general population but also in patients with pituitary disorders, such as pituitary hypersecretion conditions and hypopituitarism, predisposing to the development of osteopenia and osteoporosis. The excess and the deficits of pituitary hormones influences VD metabolism and bone metabolism, requiring careful management to prevent skeletal deterioration.

In patients with pituitary diseases, VD supplementation has shown promising results in improving bone health and reducing the incidence of fragility fractures.

Although the physio-pathological mechanisms are not fully clarified, the VD supplementation in patients with pituitary disorders should be considered in the therapeutic schedule, because of its potential protective effects in improving calcium/phosphorus and PTH metabolism, reducing bone turnover, increasing bone mineral density, and potentially reducing the risk of fragility fractures.

## Data Availability

No datasets were generated or analysed during the current study.
